# Mycological and Multiple Mycotoxin Surveillance of Sorghum and Pearl Millet Produced by Smallholder Farmers in Namibia

**DOI:** 10.1007/s00284-023-03263-7

**Published:** 2023-04-04

**Authors:** Calvin R. Kaela, Mariska Lilly, John P. Rheeder, Jane M. Misihairabgwi, Johanna F. Alberts

**Affiliations:** 1 Department of Agriculture, Cape Peninsula University of Technology, Private Bag X8, Wellington, South Africa; 2 Applied Microbial and Health Biotechnology Institute (AMHBI), Cape Peninsula University of Technology, PO Box 1906, Bellville, South Africa; 3 Department of Biotechnology and Consumer Science, Cape Peninsula University of Technology, PO Box 652, Cape Town, South Africa; 4 Department of Biochemistry and Microbiology, School of Medicine, University of Namibia, PO Box 13301, Windhoek, Namibia; 5 Department of Food Science and Technology, Cape Peninsula University of Technology, PO Box 1906, Bellville, South Africa

## Abstract

Mycological (mycotoxigenic *Fusarium* and aflatoxigenic *Aspergillus* spp.) and multiple mycotoxins [aflatoxin B_1_ (AFB_1_), fumonisin B (FB), deoxynivalenol and zearalenone] surveillance was conducted on raw whole grain sorghum (*Sorghum bicolor*) and pearl millet (*Pennisetum glaucum*) produced on smallholder farms, and processed products sold at open markets in northern Namibia. Fungal contamination was determined with morphological methods as well as with quantitative Real-Time PCR (qPCR). The concentrations of multiple mycotoxins in samples were determined with liquid chromatography tandem mass spectrometry. The incidence of mycotoxigenic *Fusarium* spp*., Aspergillus flavus* and *A. parasiticus*, as well as the concentrations of AFB_1_ and FB were significantly (*P* < 0.001) higher in the malts as compared to the raw whole grains, with *Aspergillus* spp. and AFB_1_ exhibiting the highest contamination (*P* < 0.001). None of the analysed mycotoxins were detected in the raw whole grains. Aflatoxin B_1_ above the regulatory maximum level set by the European Commission was detected in sorghum (2 of 10 samples; 20%; 3–11 µg/kg) and pearl millet (6 of 11 samples; 55%; 4–14 µg/kg) malts. Low levels of FB_1_ (6 of 10 samples; 60%; 15–245 µg/kg) were detected in sorghum malts and no FB was detected in pearl millet malts. Contamination possibly occurred postharvest, during storage, and/or transportation and processing. By critically monitoring the complete production process, the sources of contamination and critical control points could be identified and managed. Mycotoxin awareness and sustainable education will contribute to reducing mycotoxin contamination. This could ultimately contribute to food safety and security in northern Namibia where communities are exposed to carcinogenic mycotoxins in their staple diet.

**Supplementary Information:**

The online version contains supplementary material available at 10.1007/s00284-023-03263-7.

## Introduction

Sorghum (*Sorghum bicolor*) and pearl millet (*Pennisetum glaucum*) constitute half of the total cereal crop production in Africa and play an important role in the maintenance of food security [1]. Their high yield rate and adaptation to extreme environmental conditions make them suitable for agricultural utilization in most regions in Africa. As a food commodity, sorghum and pearl millet are commonly ground into flour or malted and used to prepare food and beverages, including weaning foods [1–3]. In Africa, sorghum and millet are largely subsistence food crops, but is increasingly forming the foundation of successful food and beverage industries [1]. Subsistence crops are cultivated for home consumption, i.e., for food preparation and beer brewing, as well as for informal trading.

Contamination of staple grains with mycotoxigenic fungi and mycotoxins occurs in many regions of the world, impacting negatively on food security, crop quality and trade [4, 5]. Mycotoxigenic fungi can infiltrate deep into sorghum and pearl millet matrices and produce mycotoxins during the pre-harvest, storage, transportation, processing, and marketing stages. Toxicologically significant mycotoxins include aflatoxin B_1_ (AFB_1_) produced by *Aspergillus* spp., and fumonisin B_1_ (FB_1_), fumonisin B_2_ (FB_2_), fumonisin B_3_ (FB_3_), deoxynivalenol (DON) and zearalenone (ZEA) produced by *Fusarium* spp. [3, 4]. These toxins cause a variety of biochemical effects, including carcinogenic, mutagenic, teratogenic, estrogenic, neurotoxic, hepatotoxic, nephrotoxic, cytotoxic, and immunosuppressive conditions [6]. AFB_1_ is mainly produced by *A. flavus* and *A. parasiticus.* After ingestion, AFB_1_ forms DNA adducts which initiates carcinogenesis and can work synergistically with hepatitis B virus [7]. It poses a serious threat to human and animal health by causing hepatotoxicity, teratogenicity, immunotoxicity as well as liver cancer, and is classified a Group 1 carcinogen by the International Agency for Research on Cancer (IARC) [8, 9]. The fumonisins are mainly produced by *F. verticillioides* and *F. proliferatum.* FB causes a decrease in complex sphingolipids, glycerophospholipids, and cholesterol, which are essential for cell membrane integrity, resulting in possible intestinal epithelial cell proliferation, disruption of cytokine production and modulation of intestinal barrier function [10, 11]. FB has been associated with neural tube defects, stunting in children and oesophageal cancer, and is classified a Group 2B carcinogen [8]. DON, a vomitoxin, is produced by fungi belonging to the *F. graminearum* spp. complex [12]. It causes intestinal barrier impairment and immunostimulatory effects in low doses in animals and emesis, reduction in feed conversion rate, and immunosuppression in high doses. Contamination by ZEA is mainly caused by *F. graminearum*, *F. equiseti*, *F. culmorum*, *F. cerealis* and *F. semitectum.* ZEA is an estrogenic mycotoxin affecting male and female reproductive systems [13]. Chronic exposure to mycotoxins, such as DON or DON and FB in combination, has been suggested to modulate child growth by inducing a poor appetite, gut impairment, inflammatory diarrhoea, decreased nutrient absorption and systemic immune activation [10, 14]. Co-exposure to multiple mycotoxins can have additive effects, contributing to existing health conditions and disease burden [10].

Previous reviews on mycotoxin contamination of sorghum and millet in Africa concentrated primarily on Central, Eastern and Western Africa [15]. Co-occurrence of AFs and FBs has been documented in sorghum and pearl millet from smallholder farmers under the direction of the International Institute for Tropical Agriculture, Nigeria. A surveillance study evaluating the levels of multiple mycotoxins in sorghum from Burkina Faso, Ethiopia, Mali, and Sudan resulted in 33% of 1533 samples contaminated with at least one of the AFs and FBs, sterigmatocystin, *Alternaria* toxins, ochratoxin A and ZEA [16]. Only a few reports have been documented on sorghum and millet in South Africa [17, 18], Botswana, Lesotho, Malawi, Mozambique, Zambia, and Zimbabwe [19]. Several *Fusarium* spp. have been isolated from whole grain sorghum in South Africa with *Fusarium verticillioides*, *F. proliferatum* and the *F. graminearum* spp. complex representing the main mycotoxigenic fungi [18]. Data indicated that the contamination levels of *Fusarium* and *Aspergillus* spp. and their mycotoxins do not pose a threat to the production of commercial sorghum in South Africa [17]. *F. verticillioides* and *F. nygamai* are the main fungi contaminating sorghum and millet in Lesotho and Zimbabwe [20]. In Gaborone, Botswana, 46 traditional sorghum malt, wort, and beer samples were collected from villages [21]. *F. verticillioide*s contamination was detected in 63% samples and *Aspergillus flavus* in 37%. AFs were not detected, whilst FB_1_ was detected in three malt samples (47–1316 µg/kg), and ZEA in malt (102–2213 µg/kg), wort (26–285 µg/L) and beer (20–201 µg/L) samples, respectively. There are no reports available on the occurrence of fungi and mycotoxins in raw whole grain sorghum and pearl millet in Namibia, contamination that occurs during storage, and limited reports on processed grains. In Namibia, mycotoxins below the regulatory level of the European Union (EU) were detected in pearl millet meals [3], while AFB_1_ above the EU regulatory level was detected in sorghum malts used for brewing of *oshikundu* [3], *omalodu* and *otombo* [22]. There remain a huge knowledge gap concerning fungal and mycotoxin contamination in raw staple grains produced by smallholder farmers in Namibia, and the effects of storage and processing.

Many countries have instituted risk management practices by setting regulatory maximum levels (MLs) for mycotoxins in unprocessed staple grains intended for direct human consumption, and for processed grains [5]. AFB_1_, FB_1_ and FB_2_ are the most important mycotoxins contaminating staple grains and are regulated worldwide [5]. Strict regulation of mycotoxin levels in food exist in high-income countries with high levels of food safety control to guard against the harmful effects on human health [5]. In low-income countries, mycotoxin regulations are often absent or partially implemented, leading to circumstances where mycotoxin exposures are high. In many African countries, there are large subsistence farming populations reliant on subsistence grains as their primary staple food, consuming relatively large amounts compared to urban societies [23]. Despite the reported high dietary levels of mycotoxins, legislation for their control is absent in most countries in southern Africa [19]. When last surveyed, only a few countries in Africa have mycotoxin regulations, which are primarily linked to aflatoxin exposure in the most common dietary staples (Table S1) [24, 25]. Populations that are worst affected include smallholder farmers, where mono-cereal crops are harvested and locally consumed [5]. Subsistence-grown grains can be heavily contaminated and, being consumed in large amounts, results in high exposures and consequently raises health concerns [5, 23]. Smallholder farmers in northern Namibia utilize undiversified diets due to drought conditions and are heavily reliant on sorghum and pearl millet as a staple food [3]. Relevant geographical areas include the Oshana (production of sorghum and pearl millet) and Kavango (mainly production of pearl millet) regions. Locally, the raw and processed grains are sold at open markets.

The current study determined the incidence of mycotoxigenic *Fusarium* and aflatoxigenic *Aspergillus* spp., and the levels of multiple mycotoxins (AFB_1_, FB_1_, FB_2_, FB_3_, DON and ZEA) in raw whole grain sorghum and pearl millet collected from smallholder farms and processed products sold at local markets in the Oshana region of northern Namibia. Morphological as well as molecular techniques using species-specific primers and quantitative Real-time polymerase chain reaction (qPCR) were used to determine the incidence of the fungi. The concentrations of multiple mycotoxins in samples were determined with liquid chromatography tandem mass spectrometry (LC–MS/MS).

## Materials and Methods

### Field Study and Sampling of Sorghum and Pearl Millet

**Fig. 1:**
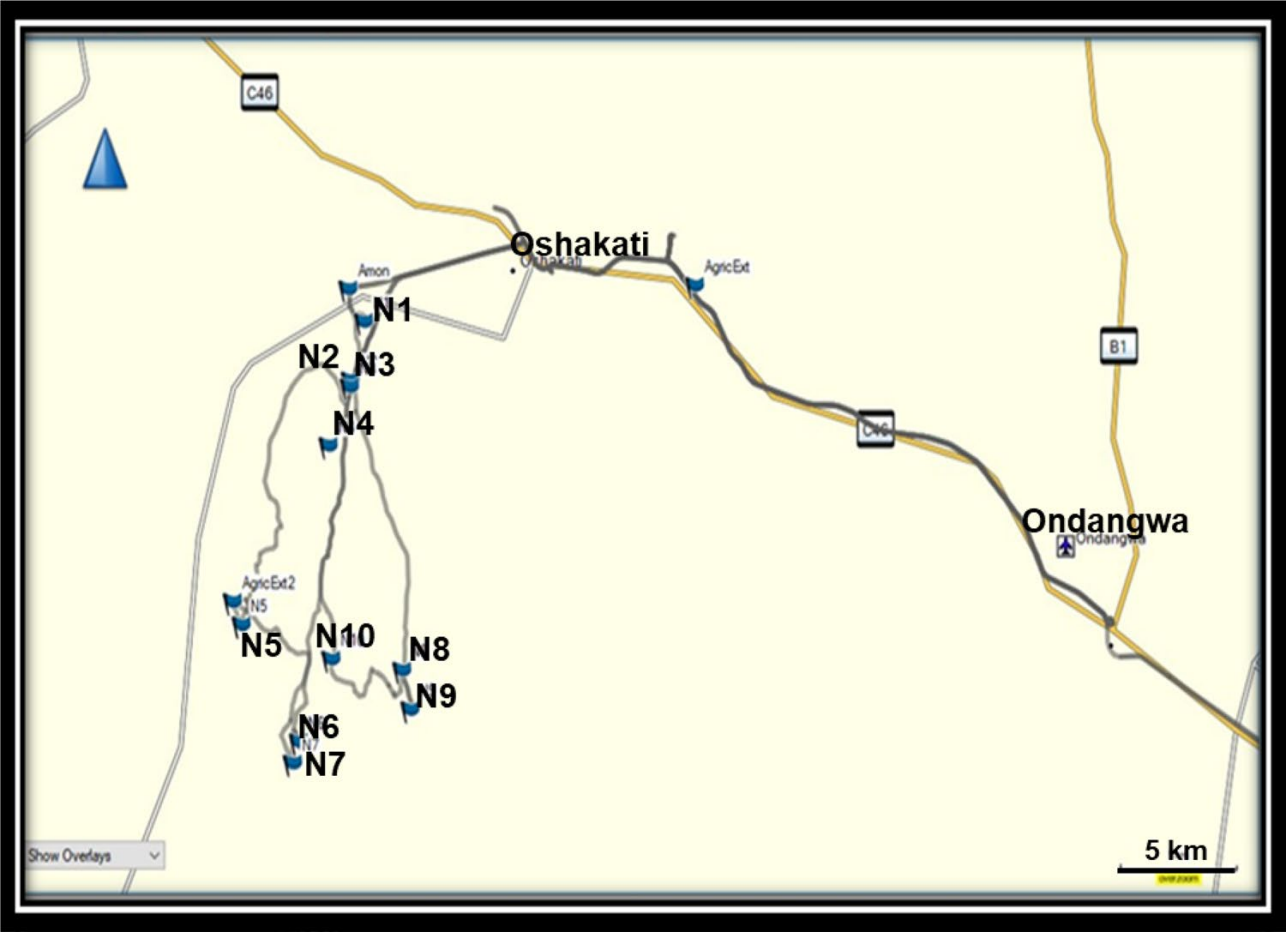
Geographical map of Namibia indicating smallholder farmer sampling sites N1-N10 near Oshakati, northern Namibia (Google Maps 2020; Photo credit: JP Rheeder)

Raw whole grain sorghum and pearl millet samples (± 2 kg) were collected postharvest, prior to storage and processing, from 10 randomly selected smallholder farms in Oshakati in the Oshana region of northern Namibia during July 2018 (Table 1). Processed samples (malted sorghum and pearl millet) were obtained from 12 and 9 randomly selected vending stalls from the Oshakati and Ondangwa open markets, respectively (Table 1). The Oshakati smallholder communal farmers service both the Oshakati and Ondangwa open markets. Figure 1 depicts a geographical map of the smallholder farming sampling sites N1-N10 near Oshakati, as determined with GPS. Standardised sampling protocols adapted from “The *Fusarium* Laboratory Manual” [26] were followed. Labelling of the samples was done according to procedure described by Safrinet [27]. The first three letters in the sample code (NAM) denoted the locality. The numeric value in the sample code represented the number of the sampling site. The last letter denotes the substratum (“S” for sorghum and M” for pearl millet). Uncontaminated (control) sorghum and pearl millet were obtained from the Mycotoxin analysis laboratory of the Southern Africa Grain Laboratory (SAGL; South Africa). To confirm the purity of the control sorghum and pearl millet samples, they were plated out onto potato dextrose agar (PDA, Lasec Group Cat no. SKU MNCM0018) to determine fungal contamination and analyzed with LC–MS/MS to determine the presence of mycotoxins. The original grain samples were stored at 4 °C, while a 200 g subsample of each original was ground to a fine meal and stored at − 20 °C for molecular and mycotoxin analyses. Mycological analysis on the original samples was concluded within four months after collection.

**Table 1:** Sampling locations of (a) raw whole grain and (b) processed sorghum and pearl millet sampled from smallholder farms and open markets in Oshakati and Ondangwa, northern Namibia

(a) Raw whole grain sorghum and pearl millet			(b) Processed sorghum and pearl millet			
Sample code	Sampling locations^a^	Sample description	Sample code	Sample description	Sample type	Sampling locations
NAM-1S	N1	Sorghum	NAM-11S	Sorghum	Malt	OSH M
NAM-2S	N2	Sorghum	NAM-12S	Sorghum	Malt	OSH M
NAM-3S	N3	Sorghum	NAM-13S	Sorghum	Malt	OSH M
NAM-4S	N4	Sorghum	NAM-14S	Sorghum	Malt	OSH M
NAM-5S	N5	Sorghum	NAM-15S	Sorghum	Malt	OSH M
NAM-6S	N6	Sorghum	NAM-16S	Sorghum	Malt	OSH M
NAM-7S	N7	Sorghum	NAM-24S	Sorghum	Malt	ONDW M
NAM-8S	N8	Sorghum	NAM-25S	Sorghum	Malt	ONDW M
NAM-9S	N9	Sorghum	NAM-26S	Sorghum	Malt	ONDW M
NAM-10S	N10	Sorghum	NAM-27S	Sorghum	Malt	ONDW M
NAM-1M	N1	Pearl millet	NAM-18M	Pearl millet	Malt	OSH M
NAM-2M	N2	Pearl millet	NAM-19M	Pearl millet	Malt	OSH M
NAM-3M	N3	Pearl millet	NAM-20M	Pearl millet	Malt	OSH M
NAM-4M	N4	Pearl millet	NAM-21M	Pearl millet	Malt	OSH M
NAM-5M	N5	Pearl millet	NAM-22M	Pearl millet	Malt	OSH M
NAM-6M	N6	Pearl millet	NAM-23M	Pearl millet	Malt	OSH M
NAM-7M	N7	Pearl millet	NAM-29M	Pearl millet	Malt	ONDW M
NAM-8M	N8	Pearl millet	NAM-30M	Pearl millet	Malt	ONDW M
NAM-9M	N9	Pearl millet	NAM-31M	Pearl millet	Malt	ONDW M
NAM-10M	N10	Pearl millet	NAM-32M	Pearl millet	Malt	ONDW M
–	–	–	NAM-33M	Pearl millet	Malt	ONDW M

^a^Sampling locations of smallholder farms (Fig. 1); *OSH M* Oshakati market, *ONDW M* Ondangwa market

### Morphological Determination of the Incidence of Mycotoxigenic *Fusarium *and Aflatoxigenic *Aspergillus* spp. in Sorghum and Pearl Millet Samples

#### Isolation and Enumeration of the Fungi from Raw Whole Grain Samples

##### *Fusarium* spp.

Subsamples of whole grain sorghum and pearl millet kernels (100 g) were surface-sterilised and rinsed with sterile distilled water [28]. One hundred kernels from each sample were plated out, five kernels per Petri dish, on modified Malt extract agar (MEA, Merck Cat. no. 105398) containing 150 mg/L novobiocin (Sigma-Aldrich, Merck Cat. no. CDS020662) and incubated at 25 °C for 10–14 days. *Fusarium* spp. that developed from the kernels were identified according to their morphological characteristics [29, 30].

##### *Aspergillus* spp.

A subsample of kernels was surface sterilized, plated onto *Aspergillus* differentiation agar (AFPA, Merck Cat. no. 17121) and incubated at 30 °C for 5 days. The presence of a yellow-orange pigment visible at the bottom of the agar plates was used to determine the presence of *A. flavus* and/or *A. parasiticus* in samples [31].

#### Isolation and Enumeration of the Fungi in Processed Samples

Contamination of the samples with mycotoxigenic *Fusarium* and aflatoxigenic *Aspergillus* spp. was determined by the dilution plate method [26]. For detection of *Fusarium* spp., dilutions were plated out on Van Wyk’s Fusarium selective medium [32] and for *A. flavus* and *A. parasiticus* on AFPA. The number of colony forming units per gram sample (cfu/g sample) were recorded.

### Molecular Identification and Quantification of Mycotoxigenic *Fusarium *and Aflatoxigenic *Aspergillus* spp. in Sorghum and Pearl Millet Samples

#### Fungal Reference Strains

*Fusarium* spp. reference strains were obtained from the Applied Microbial and Health Biotechnology Institute (AMHBI), Cape Peninsula University of Technology (CPUT), South Africa (*F. verticillioides* MRC 826, *F. proliferatum* MRC 8550 and *F. graminearum* MRC 6010). *Aspergillus* reference strains were obtained from AMHBI, CPUT [*A. parasiticus* (0200), *A. flavus* (0645 and 3954)] and from the American Type Culture Collection (ATCC, Virginia, USA) [*A. parasiticus* (CBS100926 AP1, CBS103.57 AP2 and CBS571.65 AP3) and *A. flavus* (CBS100927 AF1, CBS100.45 AF2 and CBS114062 AF3)].

#### Extraction of DNA from *Fusarium *and *Aspergillus* spp. Reference Cultures, Sorghum, and Pearl Millet Samples

Fungi were cultured in 100 ml Potato dextrose broth (PDB, Merck Cat. no. P6685) in 250 ml Erlenmeyer flasks at 26 °C on a rotary shaker for 14 days. The mycelium was harvested by filtration using a sterilized muslin cloth, and ground to a powder in liquid nitrogen with a mortar and pestle. Raw and processed sorghum and pearl millet samples (20 g) were ground to a powder using a laboratory mill (C and N laboratory mill, size 8, Christy and Norris Ltd. Engineers, Chelmsford, England). Prior to DNA extraction, the sample powder was further homogenized into a fine powder in liquid nitrogen using a mortar and pestle.

Total DNA was extracted from each sample (2 g) using a DNeasy Plant Mini Kit (Qiagen Cat. no. 69104) according to the procedure supplied by the manufacturer. The procedure was modified by the addition of 10 ml cetyltrimethylammonium bromide (CTAB)/polyvinyl-pyrrolidone (PVP) lysis buffer [33], 40 µl Proteinase K (10 mg/ml) and incubation for 2 h at 65 °C on a rotary shaker (200 rpm). The 500 µl flow through from the QIAshredder column in the DNeasy kit was transferred to a new 2 ml Eppendorf tube and an equal volume of phenol:chloroform:isoamylalcohol (P:C:I) (25:24:1; v/v) added, and the sample centrifuged at 14,000 rpm for 20 min. The top layer (450 µl) was transferred into a new 2 ml Eppendorf and an equal volume of chloroform/isoamylalcohol (24:1; v/v) (CI) added, mixed and centrifuge at 14,000 rpm for 20 min. This was done twice. The supernatant (350 µl) was transferred to a new 2 ml Eppendorf tube and continued with step 13 in the DNeasy kit protocol according to the manufacturer’s instructions. A Nanodrop 2000 Spectrophotometer (ThermoFisher Scientific, Inqaba Biotechnical Industries) was used to determine the DNA concentrations and purities by comparing the absorbance ratios A260/A280 and A260/A230. The quality of the DNA was visualized on a 0.8% agarose gel laced with ethidium bromide (5 µl) and run in 1 × Tris–acetate and EDTA (TAE) buffer electrophoresis at 70 V for 45 min [33]. A molecular weight marker, λ Hind III (ThermoFisher Scientific Cat. no. SM0101) (3 µl), containing a loading dye (2 µl), was included. Genomic DNA samples were diluted to a final concentration of 30 ng/µl and stored at − 20 °C until analysed with qPCR.

### Quantitative Real-Time PCR

#### Primer Sequences

The species-specific primer sequences used for identification and quantification of *Fusarium* and *Aspergillus* spp. DNA are listed in Table S2. The *Fusarium* spp. primers were adapted from Nicolaisen et al. [34]. The primer design was based on the alignments of the elongation factor 1-alpha (*EF1α*) gene. The *Aspergillus* spp. primers were designed based on the sequence alignments of the internal transcribed spacer 2 (ITS2) region of the DNA of several strains from different origins, retrieved from nucleotide databases [35].

#### Optimization of qPCR Conditions

Quantitative Real-time PCR was performed with a CFX96 Real-time PCR detection system (Bio-Rad, California, USA). The optimum conditions for qPCR were determined by running trial and error experiments and modifying the conditions in the protocols by changing the annealing temperatures [34–36]. Optimization reactions were carried out separately on three reference strains of *Fusarium* spp. (*F. verticillioides* MRC 826*, F. proliferatum* MRC 8550 and *F. graminearum* MRC 6010) and on two *Aspergillus* spp. reference strains (*A. parasiticus* AP1 and *A. flavus* AF3) using control sorghum and pearl millet matrixes. DNA standard curves were prepared by diluting the respective fungal gDNA (2.5 ng/μl) in control sorghum and pearl millet gDNA (30 ng/μl), to obtain a four-fold dilution series. Standard curves, including a range of gDNA concentrations, were analysed to confirm acceptable ranges of the performance parameters [37].

#### Quantification of Mycotoxigenic *Fusarium *and Aflatoxigenic *Aspergillus* spp. DNA with qPCR

Quantitative Real-time PCR assays were carried out in triplicate in a total volume of 25 μl consisting of 12.5 μl 2 × SsoAdvanced universal SYBR Green Supermix (Bio-Rad Cat. no. 1725270), 250 nM of each primer and 2 μl template DNA (30 ng/μl). The assays were performed in 96-well plates using the protocols described in Table S3. Each 96-well plate included standard curve, negative control, and non-template control samples. The criteria for acceptance followed were according to the Minimum Information for Publication of Quantitative Real-Time PCR Experiments (MIQE) Guidelines [37]. Parameters included annealing temperatures, % efficiency, correlation coefficients (R^2^) and slopes (M). Quantification was conducted using the qBase + software (Bio-Rad), and the cycle threshold (Ct) values of each maize sample plotted against the logarithm of the known DNA concentrations starting quantity of standard template for each dilution to obtain the amount of fungal DNA concentration in the starting material.

### Detection and Quantification of Multiple Mycotoxins with LC–MS/MS

#### Analytical Standards

Analytical standards of FB_1_, FB_2_ and FB_3_ (purity ≥ 97%) were obtained from the Mycotoxicology Research Group of the Institute of Biomedical and Microbial Biotechnology, CPUT. Analytical standards of AFB_1_ (Cat. no. A6636), ZEA (Cat. no. Z2125), and DON (Cat. no. D0156) were obtained from Sigma-Aldrich (Merck). Individual stock solutions (0.1 mg/ml) of AFB_1_ and ZEA were prepared in acetonitrile, while FB_1_, FB_2_ and FB_3_ and DON were prepared in acetonitrile-H_2_O (1:1). Working solutions in acetonitrile-H_2_O (1:1) containing (i) AFB_1_ and ZEA (200 ng/ml) individual concentrations, and (ii) FB_1_, FB_2_ and FB_3_ and DON (5 µg/ml) individual concentrations were prepared using aliquots of the stock solutions. Two-fold dilution series of the mycotoxin working solutions were prepared by utilising an extract prepared from control sorghum and pearl millet, respectively. The matrix-matched standards were used to compensate for matrix effects in the analysis.

#### Extraction Method

Multiple mycotoxins were extracted from raw whole grain and processed sorghum and pearl millet samples following the method described by Alberts et al. [38]. Extraction solvent [methanol: acetonitrile: water; (25:25:50); 100 ml] was added to ground samples (10 g) and the mixture placed on a rotary shaker for 30 min at 80 rpm. The extracts were centrifuged at 4000×*g* for 10 min at 4 °C in a refrigerated Sorvall RC-3B centrifuge (DuPont, Norwalk, Connecticut, USA). The supernatant was diluted (1:1) with methanol: water (25:75), filtered (Whatman No. 4 filter paper) and analysed with LC–MS/MS. Control sorghum and pearl millet samples, FAPAS (Cat no T22110QC; The Food and Environmental Research Agency, York, England; contains DON, ZEA and NIV) and Biopure (Cat no QCM3C2; Industrial Analytical, Kyalami, South Africa; contains FB_1_, FB_2_ and FB_3_) certified quality control samples, containing mycotoxins in the expected concentration ranges, were included in each run.

#### LC–MS/MS Analyses

The mycotoxins were separated on a reversed-phase BEH C_18_ column (2.1 × 50 mm; particle size 1.7 µm; Waters, Milford, MA, USA) and analysed with positive electrospray ionisation (ESI) in the multiple reaction monitoring (MRM) mode in a Waters Acquity Ultra Performance Liquid Chromatograph (UPLC) coupled to a Waters Xevo TQ tandem quadrupole mass spectrometer [38]. Eluent A was water and eluent B was acetonitrile, both containing 0.1% formic acid. The elution gradient consisted of an initial mobile phase composition (2% B) held constant for 0.5 min, followed by a linear gradient to 40% B within 7 min and to 70% B over 3 min, followed by a 1-min wash step at 100% B and finally a 3-min column re-equilibration to 2% B for a total run time of 15 min. The flow rate of the mobile phase was 0.35 ml/min. For each compound, one precursor and two product ions were monitored, one product ion for quantification and one for confirmation (Table S4). Quantification was conducted using The TargetLynx™ Application Manager of Masslynx 4.1 (Waters Corporation, Massachusetts, USA) for sample data acquisition, processing, and reporting for quantitative results relative to analytical standard calibrations. Validation experiments were performed to confirm the accuracy of the results, as described by the United States Food and Drug Administration [39]. Validation parameters included percentage (%) recovery of the extraction method, limit of quantification (LOQ), relative standard deviation for repeatability (RSDr) and the coefficient of determination (R^2^) of the respective mycotoxin calibration curves (Table 2).

**Table 2:** Performance parameters of the analytical method for quantification of multiple mycotoxins in sorghum and pearl millet samples

Analyte	Recovery (%)	LOQ (µg/kg)	RSDr (%)	Coefficient of determination (R^2^)
Sorghum				
AFB_1_	95	2	1	0.9949
FB_1_	86	5	2	0.9940
FB_2_	68	20	4	0.9950
FB_3_	76	20	1	0.9958
DON	86	100	4	0.9975
ZEA	91	10	3	0.9938
Pearl millet				
AFB_1_	95	2	2	0.9940
FB_1_	90	2	1	0.9975
FB_2_	70	10	3	0.9951
FB_3_	80	10	2	0.9963
DON	85	100	4	0.9980
ZEA	90	10	2	0.9990

*LOQ* Limit of quantification, *RSDr* Relative standard deviation for repeatability, *AFB*_*1*_ aflatoxin B_1_, *FB*_*1*_ fumonisin B_1_, *FB*_*2*_ fumonisin B_2_, *FB*_*3*_ fumonisin B_3_, *DON* deoxynivalenol, *ZEA* zearalenone

### Statistical Analyses

The NCSS Version 11 software [40] was used for statistical analysis. Data was analysed within a generalised linear model ANOVA. P < 0.05 was used as statistical significance. Correlation coefficients® were determined using R software, version 4.02 [41].

## Results

### Morphological Determination of the Incidence of Mycotoxigenic *Fusarium *and Aflatoxigenic *Aspergillus* spp. in Raw Whole Grain and Processed Sorghum and Pearl Millet Samples

#### Raw Whole Grain Samples

Samples NAM-1, NAM-2 and NAM-3 for both sorghum and pearl millet, obtained from smallholder farming sampling sites N1, N2 and N3 near Oshakati, exhibited the highest incidence of fungal contamination (*P* < 0.001) (Table 3). The contamination frequency of *Fusarium* spp. in sorghum was 80%, with NAM-1S (42%), NAM-2S (27%) and NAM-3S (39%) exhibiting the highest percentage kernel infection (P < 0.001). The *Aspergillus* spp. were not detected in sorghum samples, which confirmed results obtained during a study on commercial sorghum produced in South Africa [17]. In the current study, the contamination frequency of *Fusarium* spp. in pearl millet was 80%, with NAM-1M (9%), NAM-2M (4%) and NAM-3M (5%) exhibiting the highest percentage kernel infection (P < 0.05). The contamination frequency of the *Aspergillus* spp. in pearl millet samples was 33% with NAM-2M (10%), NAM-3M (10%) and NAM-8M (10%) exhibiting the highest percentage kernel infection (*P* < 0.001). *Fusarium* and the *Aspergillus* spp. co-occurred in 30% of pearl millet samples.

**Table 3:** The (a) incidence of mycotoxigenic *Fusarium* and aflatoxigenic *Aspergillus* spp. expressed as percentage (%) kernel infection, in sorghum and pearl millet raw whole grain and (b) contamination expressed as colony-forming units per gram (cfu/g) in processed sorghum and pearl millet, as determined with morphological methods

(a) Raw whole grain sorghum and pearl millet			(b) Processed sorghum and pearl millet		
Sample	*Fusarium* spp. (%)	Total* A. flavus* and *A. parasiticus* (%)	Sample	*Fusarium* spp. (cfu/g)	Total* A. flavus* and *A. parasiticus* (cfu/g)
NAM-1S	42	0	NAM-11S	0	7 × 10^7^
NAM-2S	27	0	NAM-12S	6 × 10^5^	9 × 10^6^
NAM-3S	39	0	NAM-13S	2 × 10^4^	6 × 10^4^
NAM-4S	2	0	NAM-14S	7 × 10^3^	2 × 10^4^
NAM-5S	1	0	NAM-15S	4 × 10^2^	1.1 × 10^2^
NAM-6S	0	0	NAM-16S	0	1 × 10^6^
NAM-7S	0	0	NAM-24S	3 × 10^5^	3 × 10^3^
NAM-8S	2	0	NAM-25S	1 × 10^4^	2 × 10^7^
NAM-9S	14	0	NAM-26S	2 × 10^4^	2 × 10^3^
NAM-10S	5	0	NAM-27S	6 × 10^5^	9 × 10^1^
NAM-1M	9	0	NAM-18M	2.3 × 10^3^	1.1 × 10^7^
NAM-2M	4	10	NAM-19M	1.7 × 10^6^	1.2 × 10^7^
NAM-3M	5	10	NAM-20M	0	8 × 10^6^
NAM-4M	2	0	NAM-21M	0	7 × 10^6^
NAM-5M	1	0	NAM-22M	1.2 × 10^4^	5 × 10^6^
NAM-6M	0	0	NAM-23 M	7 × 10^2^	3 × 10^5^
NAM-7M	2	0	NAM-29M	5 × 10^4^	1 × 10^2^
NAM-8M	1	10	NAM-3 M	2 × 10^1^	4 × 10^1^
NAM-9M	0	0	NAM-31M	0	2 × 10^1^
NAM-10M	2	0	NAM-32M	0	4 × 10^5^
–	–	–	NAM-33 M	1 × 10^1^	5 × 10^2^

#### Processed Samples

Contamination with *Fusarium* (15 of 21 samples; 71%) and the *Aspergillus* spp. (21 of 21 samples; 100%) was detected in the malts obtained from markets in Oshakati and Ondangwa (Table 3). The incidence of the *Aspergillus* spp. was significantly (*P* < 0.05) higher as compared to *Fusarium* spp. The highest contamination levels with the *Aspergillus* spp. were observed with NAM-11S (7 × 10^7^ cfu/g), NAM-18M (1.1 × 10^7^ cfu/g), NAM-19M (1.2 × 10^7^ cfu/g) and NAM-25S (2 × 10^7^ cfu/g) (*P* < 0.05). NAM-11S, NAM-18M and NAM-19M were obtained from the Oshakati market, whereas NAM-25S was obtained from the Ondangwa market. NAM-12S (6 × 10^5^ cfu/g), NAM-19M (1.7 × 10^6^ cfu/g), NAM-24S (3 × 10^5^ cfu/g) and NAM-27S (range 6 × 10^5^ cfu/g) exhibited the highest levels of *Fusarium* spp. contamination, with the highest incidence detected in pearl millet malt (*P* < 0.01). NAM-12S and NAM-19M were obtained from the Oshakati market, and NAM-24S and NAM-27S from the Ondangwa market. *Fusarium* and *Aspergillus* spp. co-occurred in 15 of 21 samples (74%) of malts sold at open markets.

### Molecular Identification and Quantification of Mycotoxigenic *Fusarium *and Aflatoxigenic *Aspergillus* spp. in Sorghum and Pearl Millet Samples with qPCR

For optimization of the *Fusarium* qPCR reactions, the standard curves generated by applying *F. verticillioides, F. proliferatum* and *F. graminearum*, and species-specific primers showed linearity across the spectrum of the serial dilution concentrations used (Table S5). They also exhibited strong correlation coefficients, suggesting low inter-assay variability in all cases. The slope of the standard curves and the amplification efficiencies attained were within the acceptable range. To confirm the purity of the control sorghum and pearl millet samples, they were plated out onto potato PDA and analysed for fungal contamination and the results were negative in both respects. For optimization of the *Aspergillus* qPCR reactions the two standard curves created by the pairs FLAVIQ1/FLAQ2 and FLAVIQ1/PARQ2 lacked linearity across the range of concentrations used and displayed a correlation coefficient < 0.99 in all reactions. The slopes of the standard curves for *A. flavus* and *A. parasiticus* were − 2.365 and 0.039, respectively, corresponding to amplification efficiencies of 164% and 92,128 × 10^7^%. The non-template control exhibited no amplification. All the values were out of range, and this served to confirm the lack of primer specificity. Aflatoxigenic *Aspergillus* spp. could therefore not be detected and quantified in samples by qPCR.

Fungal contamination was mainly observed in the malts and with *F. verticillioides* DNA (Table 4). Only a few (2 of 20 samples; 10%) raw whole grain samples contained *F. verticillioides* DNA opposed to 19 of 21 malts (90%). The highest incidence in sorghum malts was obtained with *F. verticillioides* (9 of 10 samples; 90%), followed by *F. proliferatum* (3 of 10 samples; 30%) and *F. graminearum* (2 of 10 samples; 20%) (*P* < 0.01). 56% of the contaminated sorghum malts originated from the Oshakati market and 44% from the Ondangwa market. In pearl millet malts, the highest contamination was obtained with *F. verticillioides* (10 of 11 samples; 91%) followed by *F. graminearum* (2 of 11 samples; 18%) (*P* < 0.01). No *F. proliferatum* was detected in pearl millet malts. 60% and 40% of the contaminated pearl millet malts originated from the Oshakati and Ondangwa markets, respectively. No correlation was observed between the incidence of *Fusarium* spp. as determined with morphological methods and that obtained with qPCR methods.

**Table 4:** Concentrations (pg/µl) of mycotoxigenic *Fusarium* spp. DNA in (a) raw whole grain, and (b) processed sorghum and pearl millet samples as determined with qPCR

(a) Raw whole grain sorghum and pearl millet				(b) Processed sorghum and pearl millet			
Sample	*F. verticillioides*	*F. proliferatum*	*F. graminearum*	Sample	*F. verticillioides*	*F. proliferatum*	*F. graminearum*
NAM-1S	ND	ND	ND	NAM-11S	2.76 ± 0.05^a^	ND	0.11 ± 0.05^a^
NAM-2S	ND	ND	ND	NAM-12S	1.24 ± 0.47^b^	ND	ND
NAM-3S	ND	ND	ND	NAM-13S	9.60 ± 1.98^c^	ND	ND
NAM-4S	ND	ND	ND	NAM-14S	1.52 ± 0.17^b^	ND	ND
NAM-5S	ND	ND	ND	NAM-15S	ND	ND	ND
NAM-6S	ND	ND	ND	NAM-16S	1.49 ± 0.21^b^	ND	ND
NAM-7S	ND	ND	ND	NAM-24S	0.06 ± 2.02^d^	0.60 ± 0.50^a^	ND
NAM-8S	ND	ND	ND	NAM-25S	0.70 ± 0.18^e^	ND	ND
NAM-9S	ND	ND	ND	NAM-26S	16.69 ± 2.50^f^	0.37 ± 0.30^a^	ND
NAM-10S	ND	ND	ND	NAM-27S	4.08 ± 1.47^ g^	0.59 ± 0.60^a^	0.11 ± 0.16^a^
NAM-1M	ND	ND	ND	NAM-18M	1.31 ± 0.20^d^	ND	ND
NAM-2M	0.08 ± 0.00^a^	ND	ND	NAM-19M	0.30 ± 0.10^e^	ND	ND
NAM-3M	0.10 ± 0.00^b^	ND	ND	NAM-20M	0.66 ± 0.27^f^	ND	ND
NAM-4M	ND	ND	ND	NAM-21M	0.69 ± 0.03^f^	ND	ND
NAM-5M	ND	ND	ND	NAM-22M	0.36 ± 0.02^ g^	ND	ND
NAM-6M	ND	ND	ND	NAM-23M	0.62 ± 0.30^f^	ND	0.04 ± 0.00^a^
NAM-7M	ND	ND	ND	NAM-29M	ND	ND	ND
NAM-8M	ND	ND	ND	NAM-30M	0.11 ± 0.00^ h^	ND	ND
NAM-9M	ND	ND	ND	NAM-31M	0.60 ± 0.56^abdefh^	ND	0.02 ± 0.01^b^
NAM-10M	ND	ND	ND	NAM-32M	0.52 ± 0.18^efg^	ND	ND
–	–	–	–	NAM-33M	0.35 ± 0.16^efg^	ND	ND

Values represent means of triplicate determinations ± standard deviations. *ND* none detected. Statistical significant (*P* < 0.05) differences between rows are indicated with different letters

### Detection and Quantification of Multiple Mycotoxins in Sorghum and Pearl Millet Samples with LC–MS/MS

The LC–MS/MS performance parameters for each mycotoxin are summarised in Table 2. The performance parameters for all mycotoxins were within acceptable ranges [39]. The results indicated that the control sorghum and pearl millet contained no mycotoxins. Selectivity of the method was confirmed by the absence of co-eluting peaks. The percentage recoveries for the individual mycotoxins (68–95%) in sorghum and pearl millet remained constant between runs. Results indicated that the extraction of FB was more effective (*P* < 0.05) from pearl millet than from sorghum. Replicate analysis of samples containing known amounts of the respective mycotoxins, resulted in means within 15% from the theoretical values, confirming the accuracy of the method. Coefficients of determination (R^2^), which indicates the degree of linearity of the respective mycotoxins’ calibration curves, were > 0.993. Mycotoxin concentrations in the FAPAS and Biopure certified quality control samples were within the ranges specified by the supplier for each mycotoxin during each LC–MS/MS run.

### Contamination of Sorghum and Pearl Millet Samples with Multiple Mycotoxins

None of the analysed mycotoxins were detected in the raw whole grain samples collected postharvest, prior to storage and processing. These results correlate (R = 0.8–0.83) with the incidence of aflatoxigenic *Aspergillus* spp. (3 of 20 samples; 15%) detected with morphological methods (Table 3) and *Fusarium* spp. DNA determined with qPCR in raw whole grain samples, i.e. *F. verticillioides* (2 of 20 samples; 8%)*, F. proliferatum* (0 of 20 samples) and *F. graminearum* (0 of 20 samples) (Table 4). With regards to the mycotoxin levels in processed samples, AFB_1_ was detected in sorghum malts (2 of 10 samples; 20%; 3–11 µg/kg) and pearl millet malts (6 of 11 samples; 55%; 4–14 µg/kg) (Table 5). These results correlated (R = 0.99) with the incidence of *Aspergillus* spp. in sorghum malts (10 of 10 samples; 100%) and pearl millet malts (10 of 10 samples, 100%) as determined with morphological methods (Table 3). FB was not detected in pearl millet malts (Table 5). FB_1_ (6 of 10 samples; 60%; 15–245 µg/kg) and FB_2_ (1 of 10 samples; 10%; 42 µg/kg) were present in the sorghum malts, which correlated (R = 0.88) with the incidence of *F. verticillioides* DNA in sorghum (9 of 10 samples; 90%) as determined with qPCR (Table 4). *F. proliferatum* DNA was detected in sorghum malts (3 of 10 samples; 30%), but not in pearl millet malts. One of the sorghum malts contained ZEA (3184 µg/kg) (Table 5). No detectable levels of DON were present in the malt samples. No *F. graminearum* DNA was detected in malt samples (Table 4).

**Table 5:** Concentrations (µg/kg) of multiple mycotoxins detected in processed sorghum and pearl millet samples obtained from open markets in Oshakati and Ondangwa, northern Namibia

Sample	AFB_1_	FB_1_	FB_2_	FB_3_	DON	ZEA
NAM-11S	ND	18 ± 0.00^a^	ND	ND	ND	ND
NAM-12S	11 ± 0.98^a^	ND	ND	ND	ND	ND
NAM-13S	ND	69 ± 0.25^b^	< LOQ	ND	ND	ND
NAM-14S	ND	15 ± 0.18^c^	ND	ND	ND	ND
NAM-15S	ND	ND	ND	ND	ND	ND
NAM-16S	ND	ND	ND	ND	ND	ND
NAM-24S	ND	245 ± 16^d^	42 ± 4^a^	< LOQ	ND	3184 ± 412^a^
NAM-25S	3 ± 0.15^b^	ND	ND	ND	ND	ND
NAM-26S	ND	63 ± 0.19^e^	< LOQ	< LOQ	ND	ND
NAM-27S	ND	73 ± 16^be^	< LOQ	< LOQ	ND	19 ± 7^b^
NAM-18M	6 ± 0.69^c^	ND	ND	ND	ND	ND
NAM-19M	5 ± 0.75^c^	ND	ND	ND	ND	ND
NAM-20M	14 ± 1^d^	ND	ND	ND	ND	ND
NAM-21M	4 ± 0.30^e^	ND	ND	ND	ND	ND
NAM-22M	4 ± 0.35^e^	ND	ND	ND	< LOQ	ND
NAM-23M	< LOQ	ND	ND	ND	ND	ND
NAM-29M	ND	< LOQ	ND	ND	ND	ND
NAM-30M	ND	ND	ND	ND	ND	ND
NAM-31M	ND	< LOQ	ND	ND	ND	ND
NAM-32M	4 ± 0.27^e^	ND	ND	ND	< LOQ	ND
NAM-33M	ND	ND	ND	ND	ND	ND

Values represent means ± standard deviations of three replicates. Statistical differences (*P* < 0.05) between rows are indicated with different letters. *AFB*_*1*_ aflatoxin B_1_, *FB*_*1*_ fumonisin B_1_, *FB*_*2*_ fumonisin B_2_, *FB*_*3*_ fumonisin B_3_, *DON* deoxynivalenol, *ZEA* zearalenone, *OSH M* Oshakati market, *ONDW M* Ondangwa market, *LOQ* limit of quantification, *ND* none detected

## Discussion

In Namibia, planting of sorghum and pearl millet crops takes place during November, and crops are harvested during June and July of the next year. After the growing season, crops are left to dry in the field [1, 42]. The stems are cut beneath the heads and the heads collected in harvesting baskets for further drying and threshing. As observed during the field study, threshing is performed close to the field, by hand, on hardened ground. Threshed heads are sun-dried on a threshing floor (Fig. S1) or on a raised wooden platform. Physical damage to heads exposes the powdery endosperm, thereby enhancing susceptibility to fungal infection during sun-drying, storage, and processing [1, 42]. Most households in the Oshana region store their sorghum and pearl millet for prolonged periods in traditional storage baskets (Fig. S2). The traditional granaries are large spherical woven baskets made of Mopani branches that are woven together using the bark. The internal surface is plastered using mud from ant and termite hills. The basket has a circular opening on top, which is closed by a lid and sealed by mud once loaded. Some storage baskets used in the North Central region of Namibia are made of Makalani palm leaves. Traditional methods of storage, which include the use of wood ash to guard from insect infection, could also be sources of fungal contamination [43]. Due to scarcity of trees in the Oshana region, some farmers have resorted to the use of plastic storage containers, which are commercially available.

A variety of traditional foods and beverages are prepared from sorghum. These include whole grain rice-type food, breads and pancakes, dumplings and couscous, porridges, gruels, opaque and cloudy beers, and non-alcoholic fermented beverages [1]. Throughout sub-Saharan Africa, sorghum is the grain of choice to produce traditional cloudy and opaque beers. The key ingredient of these beers is sorghum malt. Pearl millets are mainly used to prepare traditional fermented or unfermented porridges, and secondly for the brewing of traditional beers and wines [2]. Pearl millet-based gruels and steamed cakes are prepared for feeding infants and preschool children. Malted pearl millet in combination with legumes is used to prepare weaning foods. Malting is normally performed on household level and involves steeping in water (1–2 days), germination (4–5 days), and sun-drying [1, 22]. The temperature and moisture conditions during germination provide an ideal environment for fungi to proliferate, and could lead to an exponential increase in mycotoxin concentrations [22]. Milling of dried malts is traditionally performed by pounding with wooden pestles in a traditional mill until the grains are completely pulverized [22]. Some malts are transported and sold at open markets in urban areas or prepared for brewing at shebeens.

Limited information is available on the occurrence of mycotoxigenic fungi and mycotoxins along the complete sorghum and pearl millet production chain in Namibia. The present study addressed the occurrence of the main mycotoxin-producing fungi, i.e. mycotoxigenic *Fusarium* and aflatoxigenic *Aspergillus* spp. in the Oshana region of northern Namibia, mainly focussing on household grain and processed grain sold on open markets of Ondangwa and Oshakati. Samples were collected during a field study to Oshakati and Ondangwa during 2018. Traditional morphological methods as well as a validated qPCR method were used for the detection and quantification of *Fusarium* and *Aspergillus* spp. in the sorghum and pearl millet samples. A validated LC–MS/MS method was used to determine the concentrations of multiple mycotoxins in samples.

No correlation existed between the incidence of *Fusarium* spp. in samples obtained with morphological methods and that determined with qPCR. Most members of the *Fusarium* genus are morphologically similar or are cryptic species [44]. This makes it increasingly challenging and inaccurate to rely only on morphological features for identification [26, 44]. Traditional methods for identification and characterization of mycotoxigenic fungi are currently complemented with molecular based approaches such as PCR [45]. PCR-based genotyping based on sequence variability and the presence of certain genes such as *EF1α*, translation elongation factor 1-alpha (*TEF1-α*), *IGS*, and mycotoxin biosynthetic genes such as *FUM1*, *TRI13* and *TRI17* has become useful and more reliable fungal identification methods [46]. In this study, the use of species-specific primers allowed the detection and quantification of mycotoxigenic *Fusarium* spp. in samples. It should, however, be noted that qPCR can only be applied to grain samples to detect and quantify fungal species (i) that are known, (ii) for which species-specific primers are available, and (iii) for which species-specific primers have been optimized for the relevant grain matrix. It should therefore be used to complement mycological methods, to ensure that the correct fungal species are targeted.

High quality DNA was extracted from sorghum and pearl millet samples as well as from liquid cultures of reference *Fusarium* and *Aspergillus* spp. The DNA was extracted from 2 g of each sample. This was done to reduce the errors caused by non-uniform distribution of *Fusarium* spp. in samples. Although a higher amount of plant tissue might be desirable, it could saturate the mini extraction column and have a negative effect on the efficacy of DNA extractions. The DNA reading on the Thermo Scientific Nanodrop 2000 Spectrophotometer showed all the DNA samples used in these experiments have high molecular weight fragments with an A260/280 ratio between 1.8 and 2.0. To ensure that both the fungal and plant cell walls were properly broken and a high yield of gDNA was obtained during extraction, a liquid nitrogen homogenize step was included in the protocol for both *Fusarium* and *Aspergillus* spp. strains as well as for the sorghum and pearl millet samples. Phenolics are considered as the main contaminants in plant and fungal DNA preparation [47]. Phenolics, being strong oxidizing agents, decrease the yield and purity of DNA by binding covalently to the isolated DNA, thus inhibiting further enzymatic reactions of DNA, such as PCRs. The CTAB and the addition of PVP, an antioxidant, to the extraction buffer assist in eliminating phenolics in DNA extracted from plants and fungi. To ensure complete removal of phenols from the fungal gDNA, PCI and CI steps were added as modifications to the protocol provided by the manufacturer. Unfortunately, the *Aspergillus* spp. could not be detected in the sorghum and pearl millet samples, due to primer non-specificity. Aflatoxigenic *Aspergillus* spp. could, however, successfully be detected and quantified with morphological methods using Aspergillus differentiation agar.

No mycotoxins were detected in any of the raw sorghum and pearl millet whole grain samples collected from 10 households of smallholder farmers in Oshakati, postharvest, prior to storage and processing. Contrary to the raw whole grain samples, the processed samples contained mycotoxins (AFB_1_, FB and ZEA) of which MLs in food have been set for many countries by the Codex Alimentarius Commission [24, 48] and the European Commission (EC) [49]. Two of 10 (20%) sorghum and 6 of 11 (55%) pearl millet malts contained AFB_1_ above the ML of 2 µg/kg for processed cereals set by the EC [49]. A high correlation was observed between the incidences of *Aspergillus* spp. and AFB_1_ contamination. Comparable levels of AFB_1_ and FB_1_ were previously detected in sorghum malts used for the brewing of *oshikundu* [3], *amaludo* [22] and *otombo* in Namibia. AFB_1_ (4.5 ± 5.5 µg/kg) and FB_1_ (28.2 ± 33.3 μg/kg) were detected in sorghum malts used for brewing of *oshikundu* [3]. AFB_1_ (2.87–15.1 µg/kg) and FB_1_ (29.12–61.4 µg/kg) were detected in sorghum malts used for brewing *omalodu* and *otombo* [22]. In the current study, one of the sorghum malts contained ZEA above the MLs set by the EC [49] for cereal-based food and baby foods for infants and young children. Contamination by ZEA often co-occurs with DON [50]. However, there were non-detectable levels of DON contamination in the processed samples. The results of this study indicated that contamination of the processed samples occurred postharvest, possibly during storage and/or transportation and processing, due to an environment that favoured the proliferation of certain fungal species and subsequent production of mycotoxins.

Co-contamination of food and co-exposure of particularly young children to multiple mycotoxins in their staple diet have been extensively reported in certain communities in African and Latin American countries [5, 51, 52]. The Joint Food and Agricultural Organisation of the United Nations (FAO)/World Health Organisation (WHO) Expert Committee on Food Additives (JECFA) has expressed concern about the possible interaction between AFB_1_, a human genotoxin and carcinogen, and FBs, which have the potential to induce regenerative cell proliferation [53]. JECFA has determined Provisional Maximum Tolerable Daily Intake (PMTDI) levels for total FB of 2 µg/kg body weight (bw) per day [54]. It has, however, been demonstrated that the day-to-day high consumption levels of grains containing low concentrations of FB could result in PMTDI levels above the threshold of 2 µg/kg bw per day [55]. High consumption levels and chronic exposure to contaminated grains may enhance the negative health effects, especially in immunocompromised individuals [56].

## Conclusions

Raw whole grain sorghum and pearl millet produced by smallholder farmers in northern Namibia contained none of the mycotoxins analysed. Aflatoxigenic *Aspergillus* spp. were, however, detected in all sorghum and pearl millet malts. *F. verticillioides* was the predominant *Fusarium* sp. present in malts. Eight of 21 (38%) of malts contained AFB_1_ above the ML set by the EC [49]. The co-occurrence of *Fusarium* and *Aspergillus* spp. in processed sorghum and pearl millet as well as chronic exposure of the communities to AFB_1_ and FB in their staple diet is a serious concern. The malts are sold at markets and used to prepare a variety of food and beverages, including weaning foods.

Risk management involving the implementation of control methods to reduce the levels of mycotoxins in sorghum and pearl millet malts is essential. By critically monitoring the grain production process from planting, through to harvesting, processing, transportation, and marketing, the sources of contamination and critical control points could be identified and managed. Good agricultural management, both pre-harvest and postharvest, and the implementation of hazard analysis critical control point (HACCP) systems will assist in reducing fungal growth and mycotoxin contamination [57]. Several technological methods have been developed to manage pre- and postharvest fungal growth and mycotoxin production, i.e., methods involving clay minerals, plant extracts, antioxidants, biocontrol microorganisms and enzymes [58]. In Africa, there are limited resources and a scarcity of sophisticated technologies. The WHO [59] made recommendations for reduction of mycotoxins in staple grains applicable to rural subsistence farming communities. Community-based practical and integrated interventions are relevant and need to be implemented. This could involve peer-to-peer training to improve awareness and knowledge, dissemination of community-specific good agricultural practices, hand sorting, crushing, dehulling, washing, winnowing, and milling of grains [58], as well as hermitic storage practices [60].

This study presented a risk assessment of the contamination by mycotoxigenic fungi and mycotoxins of the staple grains, sorghum and pearl millet, consumed by smallholder farming communities in the Oshana region of northern Namibia. The results indicated that mycotoxigenic *Fusarium* and aflatoxigenic *Aspergillus* spp*.* colonize mainly processed sorghum and pearl millet with the occurrence of the carcinogenic aflatoxin and fumonisin mycotoxins. To determine the full extent of contamination of staple grains with multiple mycotoxins in Namibia, surveillance studies should be extended to more regions. These studies should be followed up with characterization of the dietary exposure of vulnerable populations by considering the consumption levels of the grains and corresponding contamination levels with multiple mycotoxins [61]. Understanding the impact of mycotoxins on human health is critical to further improve the risk management processes through monitoring, management, the development of informed policy strategies and eventually the implementation of regulations. This could ultimately contribute to food safety and security in northern Namibia where communities are exposed to multiple mycotoxins in their staple diet.

## Supplementary Information

Below is the link to the electronic supplementary material.

Supplementary file1 (PPTX 6146 KB)

Supplementary file2 (DOCX 27 KB)

## Data Availability

Available on request.
