# Supplementary material for: Mycological and Multiple Mycotoxin Surveillance of Sorghum and Pearl Millet Produced by Smallholder Farmers in Namibia
Source: Curr Microbiol. 2023 Apr 4;80(5):164. doi: 10.1007/s00284-023-03263-7 (PMC10073170; doi:10.1007/s00284-023-03263-7)
Supplement: Supplementary file 1 — Supplementary file1 (PPTX 6146 KB) [file 284_2023_3263_MOESM1_ESM.pptx]

## Slide 1
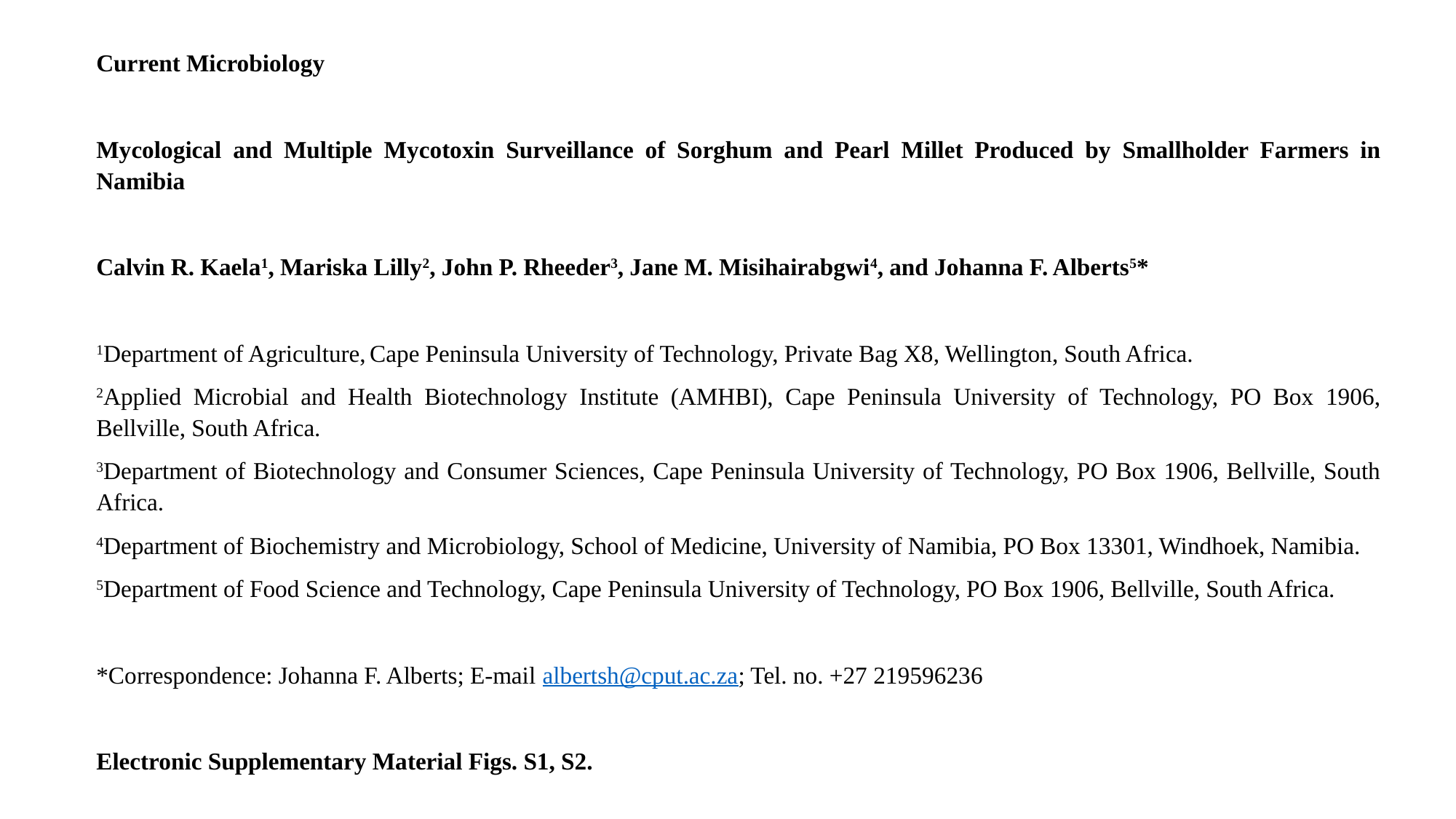

Current Microbiology
Mycological and Multiple Mycotoxin Surveillance of Sorghum and Pearl Millet Produced by Smallholder Farmers in Namibia
Calvin R. Kaela1, Mariska Lilly2, John P. Rheeder3, Jane M. Misihairabgwi4, and Johanna F. Alberts5*
1Department of Agriculture, Cape Peninsula University of Technology, Private Bag X8, Wellington, South Africa.
2Applied Microbial and Health Biotechnology Institute (AMHBI), Cape Peninsula University of Technology, PO Box 1906, Bellville, South Africa.
3Department of Biotechnology and Consumer Sciences, Cape Peninsula University of Technology, PO Box 1906, Bellville, South Africa.
4Department of Biochemistry and Microbiology, School of Medicine, University of Namibia, PO Box 13301, Windhoek, Namibia.
5Department of Food Science and Technology, Cape Peninsula University of Technology, PO Box 1906, Bellville, South Africa.
*Correspondence: Johanna F. Alberts; E-mail albertsh@cput.ac.za; Tel. no. +27 219596236
Electronic Supplementary Material Figs. S1, S2.

## Slide 2
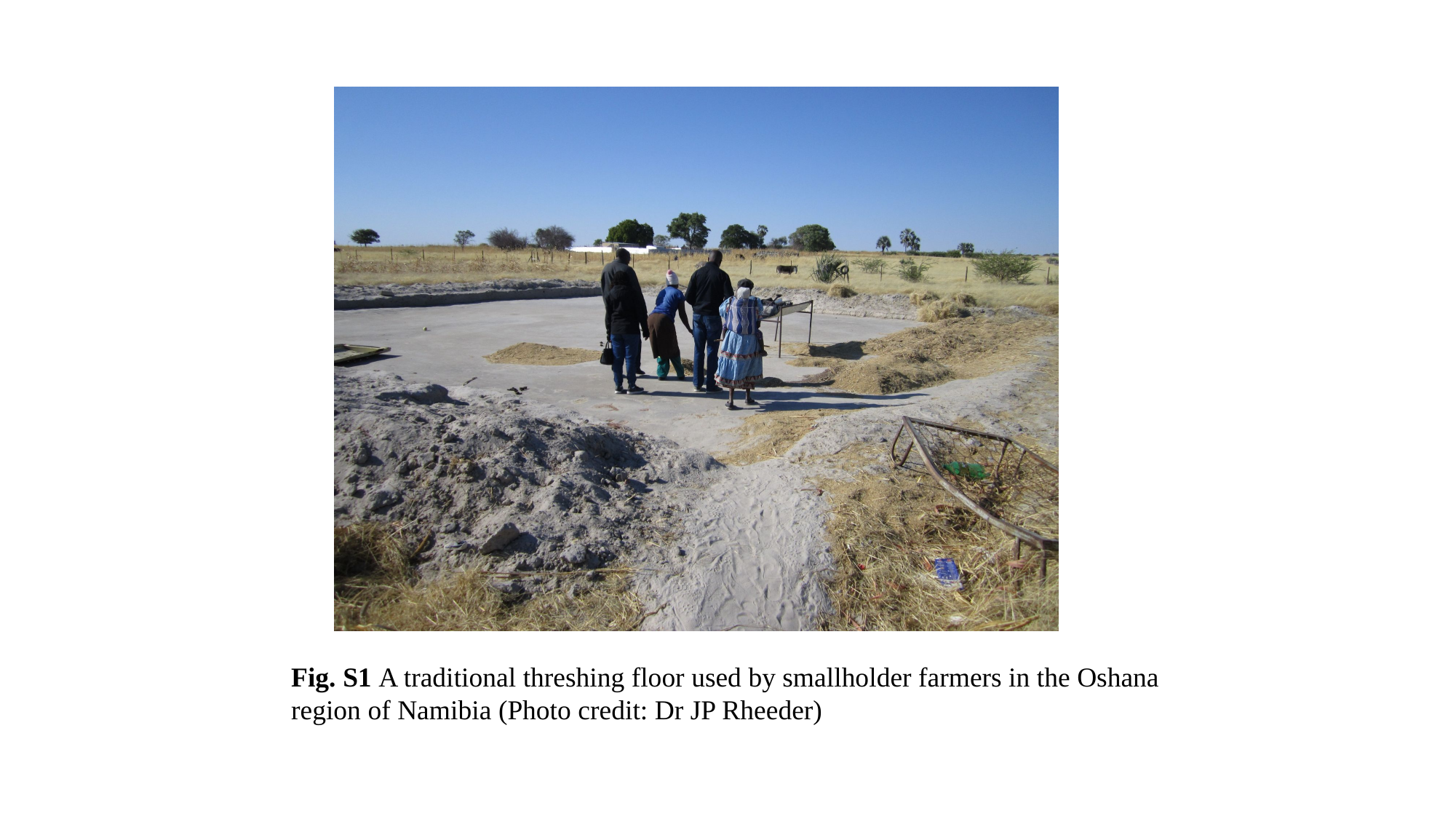

Fig. S1 A traditional threshing floor used by smallholder farmers in the Oshana region of Namibia (Photo credit: Dr JP Rheeder)

## Slide 3
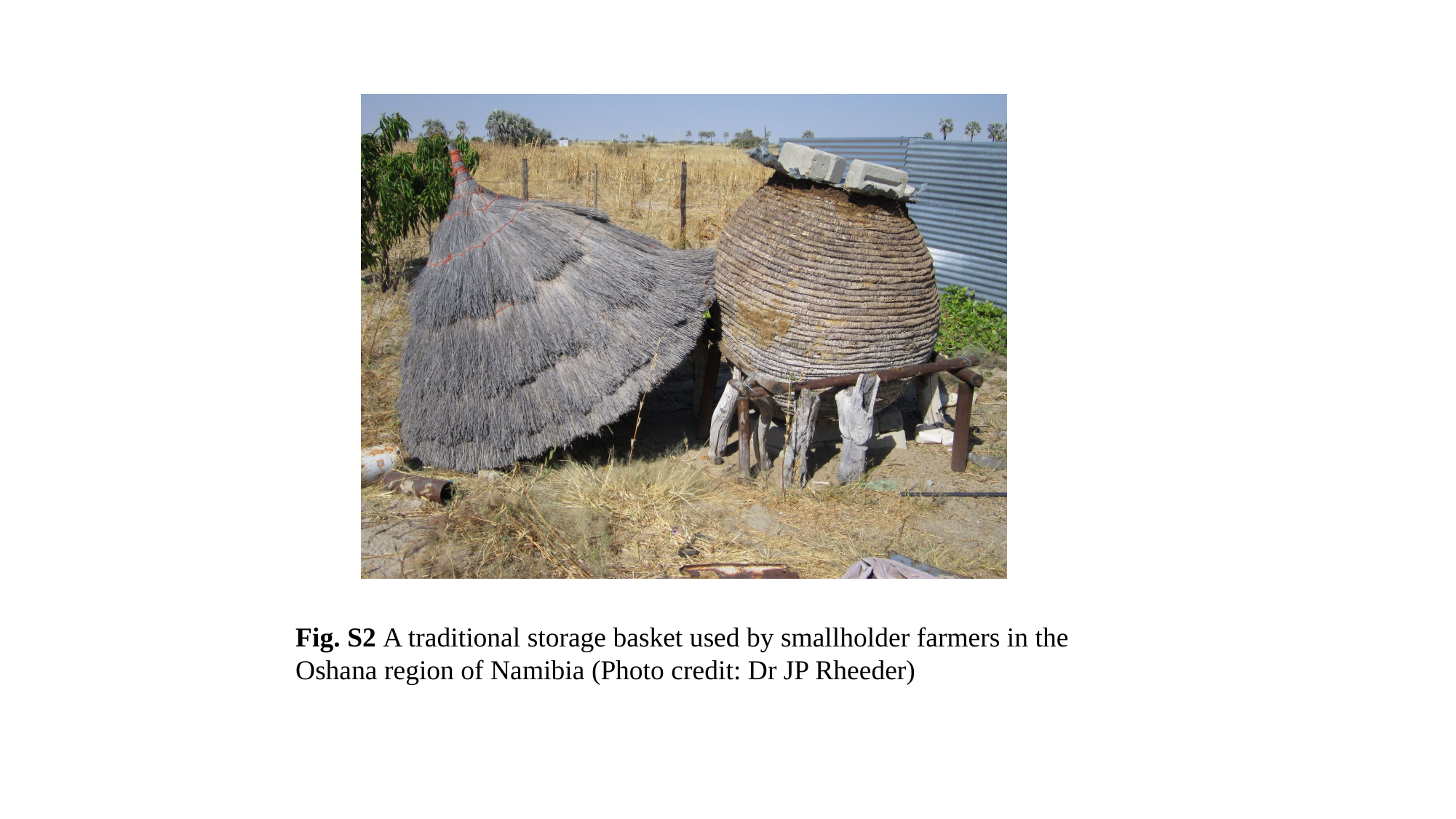

Fig. S2 A traditional storage basket used by smallholder farmers in the Oshana region of Namibia (Photo credit: Dr JP Rheeder)
