# Supplementary material for: Mycological and Multiple Mycotoxin Surveillance of Sorghum and Pearl Millet Produced by Smallholder Farmers in Namibia
Source: Curr Microbiol. 2023 Apr 4;80(5):164. doi: 10.1007/s00284-023-03263-7 (PMC10073170; doi:10.1007/s00284-023-03263-7)
Supplement: Supplementary file 2 — Supplementary file2 (DOCX 27 KB) [file 284_2023_3263_MOESM2_ESM.docx]

**Current Microbiology**

**Mycological and Multiple Mycotoxin Surveillance of Sorghum and Pearl Millet Produced by Smallholder Farmers in Namibia**

**Calvin R. Kaela^1^, Mariska Lilly^2^, John P. Rheeder^3^, Jane M. Misihairabgwi^4^, and Johanna F. Alberts^5^***

^1^Department of Agriculture, Cape Peninsula University of Technology, Private Bag X8, Wellington, South Africa.

^2^Applied Microbial and Health Biotechnology Institute (AMHBI), Cape Peninsula University of Technology, PO Box 1906, Bellville, South Africa.

^3^Department of Biotechnology and Consumer Sciences, Cape Peninsula University of Technology, PO Box 1906, Bellville, South Africa.

^4^Department of Biochemistry and Microbiology, School of Medicine, University of Namibia, PO Box 13301, Windhoek, Namibia.

^5^Department of Food Science and Technology, Cape Peninsula University of Technology, PO Box 1906, Bellville, South Africa.

*Correspondence: Johanna F. Alberts; E-mail [albertsh@cput.ac.za](mailto:albertsh@cput.ac.za); Tel. no. +27 219596236

**Electronic Supplementary Material: Tables S1, S2, S3, S4.**

**Table S1** Regulatory maximum levels for mycotoxins in cereal grains implemented by African countries [adapted from a survey on worldwide regulations of mycotoxins in food and feed conducted by the Food and Agriculture Organization of the United Nations [24,25]]

| **Country** | **Food commodity** | **Mycotoxin** | **Regulatory maximum limit (µg/kg)** |
| --- | --- | --- | --- |
| Algeria | Peanuts, nuts, cereals | AFB_1_ | 10 |
|  |  | Total AFB_1_, AFB_2_,  AFG_1_, AFG_2_ | 20 |
| Côte d’Ivoire | Straight feedstuffs | Total AFB_1_, AFB_2_, AFG_1_, AFG_2_ | 100 |
|  | Complete feedstuffs |  | 10 |
|  | Complete feedstuffs for pigs/poultry except young animals/ducks |  | 38 |
|  | Complete feedstuffs for cattle/sheep/goats |  | 75 |
|  | Complete feedstuffs for dairy cattle |  | 50 |
| Egypt | Peanuts, cereals | AFB_1_ | 5 |
|  |  | Total AFB_1_, AFB_2_,  AFG_1_, AFG_2_ | 10 |
|  | Maize | AFB_1_ | 10 |
|  |  | Total AFB_1_, AFB_2_,  AFG_1_, AFG_2_ | 20 |
|  | Animal and chicken feed | AFB_1_ | 10 |
|  |  | Total AFB_1_, AFB_2_,  AFG_1_, AFG_2_ | 20 |
|  | Edible part of nuts which require additional  treatment  before use | AFB_1_ | 5 |
|  | Cereals, cereal flours,  cereal groats and flakes |  | 2 |
| Kenya | Peanut (products) | Total AFB_1_, AFB_2_,  AFG_1_, AFG_2_ | 20 |
| Malawi | Peanuts (export) | AFB_1_ | 5 |
| Morocco | All foods |  | 10 |
|  | Peanuts, pistachio nuts,  almonds |  | 1 |
|  | Wheat meal |  | 3 |
|  | Wheat bran |  | 10 |
|  | Cereals, wheat meal  (complete) |  | 5 |
|  | Cereals | ZEA | 200 |
|  | Cereals | OTA | 30 |
|  | Simple feedstuffs | AFB_1_ | 50 |
|  | Peanuts, copra,  cottonseed, babassu, maize and their products |  | 20 |
|  | Complete feedstuffs for  cattle, sheep and goats |  | 50 |
|  | Complete feedstuffs for dairy animals |  | 5 |
|  | Complete feedstuffs for calves and lambs |  | 10 |
|  | Complete feedstuffs for pigs and poultry |  | 20 |
|  | Complementary feedstuffs for cattle, sheep and goats |  | 50 |
|  | Complementary feedstuffs for pigs and poultry |  | 30 |
| Mozambique | Peanuts |  | 10 |
| Nigeria | All foods |  | 20 |
| Senegal | Peanut products (straight feedstuffs) |  | 50 |
|  | Peanut products (feedstuff ingredients) |  | 300 |
| South Africa | All foods |  | 5 |
|  |  | Total AFB_1_, AFB_2_,  AFG_1_, AFG_2_ | 10 |
| Sudan | Wheat | OTA | 15 |
| Tanzania | Cereals, oil seeds | AFB_1_ | 5 |
|  |  | Total AFB_1_, AFB_2_,  AFG_1_, AFG_2_ | 10 |
|  | Feeds | AFB_1_ | 5 |
|  |  | Total AFB_1_, AFB_2_,  AFG_1_, AFG_2_ | 10 |
| Tunisia | All foods | AFB_1_ | 2 |
| Zimbabwe | All foods |  | 5 |
|  | Groundnuts, maize,  sorghum |  | 5 |
|  |  | AFG_1_ | 4 |
|  | Poultry feed | Total AFB_1_ and AFG_1_ | 10 |

AFB_1_, aflatoxin B_1_; AFB_2_, aflatoxin B_2_, AFG_1_, aflatoxin G_1_; AFG_2_, aflatoxin G_2_; ZEA, zearalenone; OTA, ochratoxin A

**Table S2** The species-specific primer sequences used for identification and quantification of mycotoxigenic *Fusarium* and aflatoxigenic *Aspergillus* spp. in sorghum and pearl millet samples with qPCR

| Target species | Primer Name | Primer sequence (5′–3′) | Reference |
| --- | --- | --- | --- |
| *F. verticillioides* | Fver356 fwd  Fver412 rev | CGTTTCTGCCCTCTCCCA  TGCTTGACACGTGACGATGA | [34] |
| *F. proliferatum* | Fpro220 fwd  Fpro270 rev | CTTCGATCGCGCGTCCT  CACGTTTCGAATCGCAAGTG | [34] |
| *F. graminearum* | FgramB379 fwd  FgramB411 rev | CCATTCCCTGGGCGCT  CCTATTGACAGGTGGTTAGTGACTGG | [34] |
| *A. flavus* | FLAVIQ1 fwd  FLAQ2 rev | GTCGTCCCCTCTCCGG  CTGGAAAAAGATTGATTTGCG | [35] |
| *A. parasiticus* | FLAVIQ 1 fwd  PAR Q2 rev | GTCGTCCCCTCTCCGG  GAAAAAATGG TTGTTTTGCG | [35] |

Fwd, forward primer. Rev, reverse primer

**Table S3** The experimental protocols used for the detection and quantification of mycotoxigenic *Fusarium* and aflatoxigenic *Aspergillus* spp*.* in sorghum and pearl millet samples with qPCR

| Target species | qPCR cycle | | | | | | |
| --- | --- | --- | --- | --- | --- | --- | --- |
|  | **1** | **2** | **3** | **4** | **5** | **6** | **7** |
| *F. verticillioides* | 95 °C; 10 min | 95 °C; 15 s | 66 °C; 15 s | 72 °C; 15 s | 95 °C; 10 s | 72 °C; 0.05 s | 95 °C; 0.5 s |
| *F. proliferatum* | 95 °C; 10 min | 95 °C; 15 s | 66 °C; 15 s | 72 °C; 15 s | 95 °C; 10 s | 72 °C; 0.05 s | 95 °C; 0.5 s |
| *F. graminearum* | 95 °C; 10 min | 95 °C; 15 s | 72 °C; 15 s | 95 °C; 15 s | 72 °C; 0.05 s | - | 95 °C; 0.5 s |
| *A. flavus* | 95°C; 10 min | 95°C; 15 s | 60°C; 60 s | - | 95°C; 10 s | 65°C; 0.05 s | 95°C; 0.5 s |
| *A. parasiticus* | 95°C; 10 min | 95°C; 15 s | 60°C; 60 s | - | 95°C; 10 s | 65°C; 0.05 s | 95°C; 0.5 s |

Protocols were adapted and modified from the procedures described by Boutigny et al. [41] for *Fusarium* spp. and by Sardiñas et al. [35] for *Aspergillus* spp. For each reaction, 40 cycles were included

**Table S4** LC-MS/MS conditions for quantification of multiple mycotoxins in sorghum and pearl millet samples by positive ESI at capillary voltage 3.5 kV

| **Analyte** | **Cone voltage (V)** | **Precursor ion** | **Quantifier Ion (Collision energy)**  **(V)** | **Qualifier Ion (Collision energy)**  **(V)** |
| --- | --- | --- | --- | --- |
| Aflatoxin B_1_ | 50 | 313 | 285 (23) | 241 (37) |
| Fumonisin B_1_ | 50 | 722.3 | 334.3 (40) | 352.3 (38) |
| Fumonisins B_2_ and B_3_ | 50 | 706.3 | 318.3 (40) | 336.3 (40) |
| Deoxynivalenol | 35 | 397.1 | 203.2 (15) | 231.2 (12) |
| Zearalenone | 20 | 319.1 | 185.0 (23) | 187.0 (19) |

**Table S5** Optimum conditions for identification and quantification of mycotoxigenic *Fusarium* spp. in sorghum and pearl millet samples with qPCR

| Reference fungal strains | Matrix | Annealing  Temperature (˚C) | Efficiency (%) | Correlation  coefficient (R²) | Slope  (M) |
| --- | --- | --- | --- | --- | --- |
| *F. verticillioides* MRC 826 | Pearl millet | 66 | 101.0 | 0.991 | -3.298 |
| *F. verticillioides* MRC 826 | Sorghum | 60 | 106.6 | 0.992 | -3.174 |
| *F. proliferatum* MRC 8550 | Pearl millet | 64 | 106.2 | 0.992 | -3.182 |
| *F. proliferatum* MRC 8550 | Sorghum | 66 | 122.7 | 0.994 | -2.877 |
| *F. graminearum* MRC 6010 | Pearl millet | 64 | 101.3 | 0.993 | -3.292 |
| *F. graminearum* MRC 6010 | Sorghum | 62 | 117.3 | 0.993 | -2.966 |
